# Supplementary material for: Modeling glioblastoma heterogeneity as a dynamic network of cell states
Source: Mol Syst Biol. 2021 Sep 16;17(9):e10105. doi: 10.15252/msb.202010105 (PMC8444284; doi:10.15252/msb.202010105)
Supplement: Supplementary file 6 — Source Data for Figure 5 [file MSB-17-e10105-s004.zip › Figure5A_sourcedata/GSEA_3017/hallmarks_stateE.GseaPreranked.1621934677158/HALLMARK_HYPOXIA.html]

Details for gene set HALLMARK\_HYPOXIA[GSEA]

|  || Dataset | state63017 |
| Phenotype | NoPhenotypeAvailable |
| Upregulated in class | na\_pos |
| GeneSet | HALLMARK\_HYPOXIA |
| Enrichment Score (ES) | 0.470965 |
| Normalized Enrichment Score (NES) | 2.4167607 |
| Nominal p-value | 0.0 |
| FDR q-value | 0.0 |
| FWER p-Value | 0.0 |
Table: GSEA Results Summary

  

Fig 1: Enrichment plot: HALLMARK\_HYPOXIA      
 Profile of the Running ES Score & Positions of GeneSet Members on the Rank Ordered List

  

| PROBE | GENE SYMBOL | GENE\_TITLE | RANK IN GENE LIST | RANK METRIC SCORE | RUNNING ES | CORE ENRICHMENT || 1 | ANXA2 |  |  | 18 | 0.733 | 0.0620 | Yes |
| 2 | GPC1 |  |  | 50 | 0.530 | 0.0874 | Yes |
| 3 | SERPINE1 |  |  | 52 | 0.526 | 0.1447 | Yes |
| 4 | F3 |  |  | 55 | 0.506 | 0.1989 | Yes |
| 5 | HSPA5 |  |  | 73 | 0.449 | 0.2304 | Yes |
| 6 | IRS2 |  |  | 85 | 0.426 | 0.2659 | Yes |
| 7 | VLDLR |  |  | 87 | 0.419 | 0.3115 | Yes |
| 8 | CAVIN1 |  |  | 100 | 0.399 | 0.3428 | Yes |
| 9 | ATF3 |  |  | 101 | 0.397 | 0.3870 | Yes |
| 10 | PDGFB |  |  | 131 | 0.347 | 0.3942 | Yes |
| 11 | BNIP3L |  |  | 142 | 0.341 | 0.4214 | Yes |
| 12 | JUN |  |  | 172 | 0.316 | 0.4252 | Yes |
| 13 | SDC3 |  |  | 200 | 0.295 | 0.4287 | Yes |
| 14 | SDC2 |  |  | 226 | 0.280 | 0.4328 | Yes |
| 15 | GPC4 |  |  | 246 | 0.264 | 0.4416 | Yes |
| 16 | CSRP2 |  |  | 247 | 0.264 | 0.4710 | Yes |
| 17 | CA12 |  |  | 325 | -0.263 | 0.4169 | No |
| 18 | EGFR |  |  | 427 | -0.291 | 0.3400 | No |
| 19 | CCNG2 |  |  | 467 | -0.305 | 0.3317 | No |
| 20 | SAP30 |  |  | 558 | -0.339 | 0.2720 | No |
| 21 | MIF |  |  | 580 | -0.349 | 0.2881 | No |
| 22 | MAP3K1 |  |  | 702 | -0.431 | 0.2051 | No |
| 23 | BTG1 |  |  | 772 | -0.520 | 0.1883 | No |
Table: GSEA details [plain text format]

  

Fig 2: HALLMARK\_HYPOXIA: Random ES distribution      
 Gene set null distribution of ES for **HALLMARK\_HYPOXIA**
